# Editorial: The Role of Heat Shock Proteins in Neuroprotection

**DOI:** 10.3389/fphar.2020.01227

**Published:** 2020-08-06

**Authors:** Miklos Santha, Heather D. Durham, Laszlo Vigh, Chrisostomos Prodromou

**Affiliations:** ^1^Biological Research Centre, Hungarian Academy of Sciences (MTA), Szeged, Hungary; ^2^Montreal Neurological Institute, McGill University, Montreal, QC, Canada; ^3^Biochemistry and Biomedicine, University of Sussex, Brighton, United Kingdom

**Keywords:** heat shock protein, neuroprotection, neurodegeneration, chaperone, protein aggregation

This Research Topic covers an increasingly important topic on the “Role of Heat Shock Proteins in Neuroprotection”. With a growing elderly population and an ever-increasing amount of resources required to treat neurodegeneration, there is a growing demand for solutions. Chaperone systems represent the major pro-survival strategy for cells of living organisms in response to stress. Heat shock proteins are associated with neurological disease because they can suppress or promote the aggregation of misfolded toxic proteins. This Research Topic brings together a number of selected articles that show how diverse heat shock proteins impact on neurodegenerative disease.

The review by Webster et al. discusses the role that misfolding, aggregation, and aberrant accumulation of proteins play as a central component in the progression of neurodegenerative disease. While molecular chaperones are normally protective, they can also promote the stabilization of toxic protein aggregates, which can lead to neurodegenerative disease. The authors review the role by which sHsps modulate neurodegenerative disease-relevant protein aggregation.

The manuscript by Gracia et al. looks at the therapeutic potential of the Hsp90/Cdc37 interaction in neurodegenerative disease, such as Alzheimer’s, Huntington’s, and Parkinson’s. This article presents evidence that client proteins, and in particular kinases, may be differentially affected when modulating the Hsp90/Cdc37 chaperone system, depending on the strength of their interaction with this chaperone complex. Consequently, this may allow therapeutic intervention by targeting Hsp90/Cdc37-client protein complexes, which are currently underexplored.

The role of heat shock protein’s in neuroinflammation is discussed by Dukay et al. Apart from the classical molecular chaperoning of heat shock proteins, they are shown to play a role in neurological disorders by modulating neuronal survival, inflammation, and disease-specific signalling processes. Although the processes of neuro-inflammation are not yet fully understood, the authors explore the existing literature on the inflammatory function of heat-shock-proteins within the central nervous system.

Cristofani et al. then investigates the role of HSPB8 on the protein quality control system of cells. This system prevents the deleterious effects of misfolded proteins of which HSPB8 is a component. Elevated levels of HSPB8, activated by misfolded proteins through transcription, contribute to preventing the aggregation of misfolded proteins, facilitate autophagy, and enable the efficient clearance of the misfolded proteins.

In conclusion, the Research Topic brings together some important topics showing how heat shock proteins are central to many neurological disease processes and how intervention by modulating the expression of the chaperone system can be used for therapeutic treatment. It stands as an introduction to an emerging field to stimulate research on chaperones as targets for neuroprotection.

## Author Contributions 

CP wrote and all authors edited the editorial. All authors contributed to the article and approved the submitted version.

## Conflict of Interest

The authors declare that the research was conducted in the absence of any commercial or financial relationships that could be construed as a potential conflict of interest.

